# Replicator--Mutator Dynamics on Networks with Spatial Nonlocality: Theory and Applications

**DOI:** 10.21203/rs.3.rs-11222200/v1

**Published:** 2026-10-23

**Authors:** Porikshit Mondal, Ernesto Estrada

## Abstract

The replicator--mutator model provides a general framework for studying the emergence of social norms and dominant behavioral patterns in networked populations. In the traditional network formulation, mutation and observational learning operate through nearest-neighbor interactions. In realistic settings, however, agents may also be influenced by others beyond their immediate neighbors, with the strength of influence typically decaying with distance. Here we investigate how evolutionary dynamics change when these nonlocal interactions are incorporated explicitly. We introduce a generalized interaction operator based on $d$-path Laplacians, which combines interactions across all shortest-path distances. We then analyze how the range of the coupling affects evolutionary behavior on different network topologies. Our analytical results relate mutation-induced mixing and local convergence to the spectral structure of the corresponding nonlocal random-walk Laplacian. Numerical experiments show that stronger nonlocality reduces the mutation intensity required to attain high diversity and may produce an abrupt transition from behavioral flocking to an almost uniform distribution of behaviors. Thus, long-range influence promotes globalization in the sense of increased effective diversity together with greater homogeneity of behavioral frequencies.

